# Impact of Laser Intensity Noise on Dual-Comb Absolute Ranging Precision

**DOI:** 10.3390/s22155770

**Published:** 2022-08-02

**Authors:** Jiaqi Wang, Haosen Shi, Chunze Wang, Minglie Hu, Youjian Song

**Affiliations:** 1 Ultrafast Laser Laboratory, Key Laboratory of Opto-Electronic Information Science and Technology of Ministry of Education, School of Precision Instruments and Opto-Electronics Engineering, Tianjin University, Tianjin 300072, China; jiaqi1214@tju.edu.cn (J.W.); wangchunze@tju.edu.cn (C.W.); huminglie@tju.edu.cn (M.H.); 2 State Key Laboratory of Precision Spectroscopy, East China Normal University, Shanghai 200062, China; hsshi@lps.ecnu.edu.cn

**Keywords:** dual-comb absolute ranging, laser intensity noise, linear optical sampling, asynchronous optical sampling

## Abstract

Noise in mode-locked lasers has been a central issue for dual-comb metrological applications. In this work, we investigate the laser intensity noise on dual-comb absolute ranging precision. Two different dual-comb schemes based on linear optical sampling (LOS) and nonlinear asynchronous optical sampling (ASOPS) have been constructed. In the LOS scheme, the ranging precision deteriorates with the increase in laser relative intensity noise (RIN). This effect can be corrected by implementing a balanced photo-detection (BPD). In the ASOPS scheme, the experiment shows that the conversion from laser RIN to dual-comb ranging precision is negligible, making a balanced detection unnecessary for ranging precision improvement. The different manners of RIN’s impact on absolute ranging precision are attributed to the distinct cross-correlation signal patterns and the underlying time-of-flight (TOF) extraction algorithms.

## 1. Introduction

Mode-locked lasers generate equidistant discrete optical pulse trains with ultrashort pulse duration and are taken as one of the most suitable generators for optical frequency combs (OFCs) [1]. OFCs based on passively mode-locked lasers [2] have become an enabling tool for a diversity of applications, such as laser spectroscopy, optical frequency atomic clock, and microwave photonics, to name a few [3,4,5]. All of these applications benefit from the unique time-frequency properties of optical frequency combs, which characterize a femtosecond pulse train with ultralow timing jitter [6,7] in the time domain, and a ruler formed by a series of coherent spectral lines in the frequency domain. Recently, mode-locked femtosecond lasers and various OFC sources have also been applied for distance measurement applications [8,9,10,11,12,13,14] due to the unique features above. In the time domain, the retro-reflective and the local pulse trains cross-correlate and generate an ultrashort timing gate for direct time-of-flight (TOF) measurement [8,9]. In the frequency domain, uniformly spaced phase-coherent comb lines allow a multi-wavelength interferometric measurement principle [10,11]. OFCs and mode-lock lasers have achieved absolute ranging with nanometer-precision [12,14], which outperforms conventional laser ranging techniques based on continuous wave (CW) lasers and Q-switched lasers.

The absolute ranging capability based on OFCs can be further improved by adopting a dual-comb configuration [14,15,16,17,18,19,20,21,22,23,24,25,26,27,28,29,30,31,32,33]. The principle is similar to the linear optical sampling (LOS) used in optical telecommunication systems [34,35]. The slight repetition rate gap that exists between OFCs provides an optical vernier caliper, ensuring high measurement precision and extendable non-ambiguity range (NAR). The pulse train spacing of an OFC and the difference in repetition rate between combs determine the NAR and update rate, respectively. In 2009, I. Coddington et al. demonstrated a measurement precision of 5 nm within a NAR of 1.5 m by taking advantage of tightly phase-locked dual-comb interferometry. This research aims at future multi-satellite accurate and tight formation flight applications [15]. To facilitate industrial applications, a simplified dual-comb configuration has also been developed for TOF ranging only. The pulse timing is determined by the gravity center of the interferometric beat note, and sub-micrometer ranging precision can be obtained without a tight carrier-envelope phase locking. The methods for pulse envelope acquisition vary according to different optical sampling principles [36]. For linear optical sampling (LOS), the extraction of the envelope from interferometric beat notes is achieved by applying the Hilbert transform algorithm, the harmful carrier underneath the envelope is effectively removed, and the final TOF extraction can be performed by subsequent Gaussian fitting [16]. For nonlinear asynchronous optical sampling (ASOPS), the intensity cross-correlation trace is not subject to an interferometric pattern in nature, and gaussian fitting can be directly performed on the cross-correlation traces [17].

To further improve the dual-comb absolute distance measurement performance, the impact of different laser parameters has been investigated [18]. High precision absolute ranging frequently benefits from higher repetition rate femtosecond laser sources [19,20]. The repetition rate gap [21,22,23] between the two mode-locked lasers has an impact on the fringe density of interferometric beat notes, thus affecting the ranging precision indirectly [24]. The center wavelength of the overlapping part of the spectra of the two optical frequency combs indirectly affects the ranging precision in a similar manner [25]. Quantum-limited pulse timing jitter [26] has also been shown to deteriorate ranging precision, particularly in the center of each NAR. A phase distortion correction algorithm can effectively improve ranging accuracy in LOS-based ranging systems [37], while an appropriate increase in measurement speed can reduce the cumulative ranging error caused by time jitter in nonlinear ASOPS-based ranging systems [38]. Despite the above progress, the impact of laser intensity noise on ranging precision based on the LOS and ASOPS schemes has not been explored.

In this paper, we investigate the impact of laser intensity noise on dual-comb TOF distance measurement precision for both linear optical sampling (LOS) and nonlinear asynchronous optical sampling (ASOPS). In the LOS scheme, a pair of phase-locked nonlinear amplifier loop mirror (NALM) mode-locked fiber lasers are used. The interferometric beat notes between the target/reference reflected pulses and the LO are digitalized and the following Hilbert transforming algorithm is used to extract TOF. Triple outputs of the signal laser with different intensity noise [39,40] have been used to study the impact of RIN level on measurement precision. The integrated RIN of output ports 1, 2 and 3 are 0.020%, 0.023% and 0.026%, respectively. Separate distance measurements for a target with 100 mm distance at an update rate of ~2.5 kHz have been conducted by using these output ports. When a single avalanche photodetector is used, the ranging precision evaluated by Allan deviation is 26.1 μm, 31.6 μm and 36.2 μm, respectively, corresponding to the above three output ports. When a balanced photodetector is used, the ranging precision reaches ~10 μm, regardless of laser relative intensity noise (RIN). The measurement shows that balanced photo-detection (BPD) can be used to erase the impact of laser RIN and effectively improve dual-comb ranging precision. An independent dual-comb absolute ranging experiment based on nonlinear ASOPS is conducted afterward. In this experiment, sum-frequency generation between the target/reference reflected pulses and the LO results in non-interferometric optical cross-correlation (OC) signals, which are digitalized and the TOF can be directly extracted after Gaussian-envelope fitting and peak extraction. Nonlinear ASOPS based on optical cross-correlation show similar ranging precision with balanced optical cross-correlation (BOC), where the target/reference pulse timing is extracted from the zero-crossings of the BOC signals [33]. The experiment indicates that the ranging precision is not sensitive to laser RIN.

## 2. Dual-Comb Ranging Using Linear Optical Sampling

### 2.1. Design of Femtosecond Lasers

**Figure 1:**
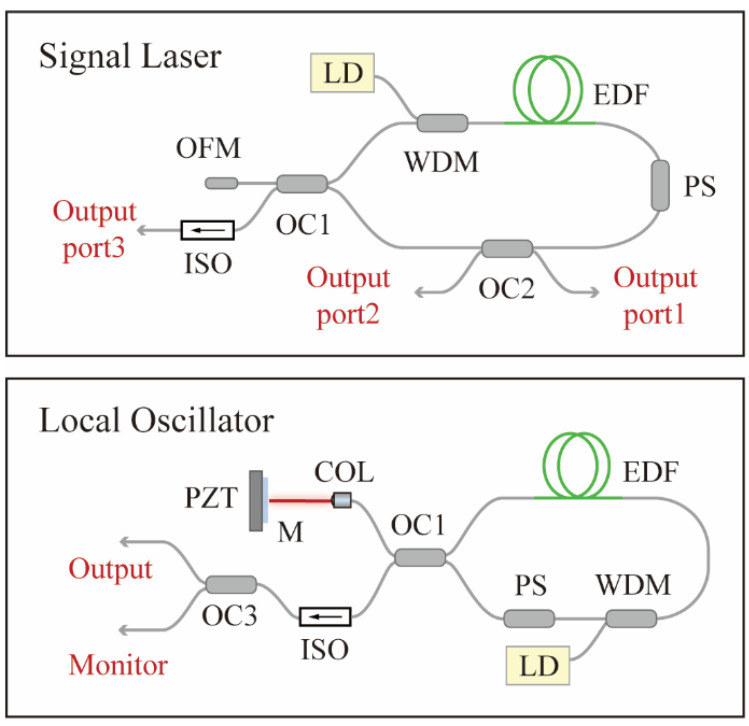
Schematic of the mode-locked lasers based on nonlinear amplifier loop mirror. COL, collimator; EDF, Er-doped gain fiber; ISO, isolator; LD, pump laser diode; M, reflective mirror; OC, optical fiber coupler; OFM, optical fiber mirror; PS, phase shifter; PZT, piezoelectric transducer; WDM, wavelength division multiplexer.

In order to study the impact of laser intensity noise on the absolute ranging based on LOS, we built an all-polarization-maintaining (all-PM) erbium-doped fiber laser mode-locked by a nonlinear amplifier loop mirror with triple outputs and a similarly structured LO laser. The schematics of the triple-output signal laser and LO laser are shown in Figure 1. The fiber loops and linear arms of two mode-locked lasers are connected by optical fiber couplers with a splitting ratio of 70/30. The end of the coupler with 70% output is used as the reflection arm, and the other end is used for output. Pump laser output from a 980 nm single-mode laser diode is coupled into a fiber loop by a 980/1550 wavelength division multiplexer (WDM). Asymmetrically placed 35 cm erbium-doped gain fiber and a non-reciprocal phase shifter ensure the self-starting performance of lasers [41]. To diversify the output parameters of the signal laser, a 90/10 splitting ratio optical coupler is added to the loop, which will provide two more outputs with different running directions. We numbered the three ports and marked them in Figure 1. The mode-locking of the signal laser is self-starting at the pump power of 650 mW and achieved stable single-pulse operation at ~100–150 mW pump power. The average output power of ports 1, 2 and 3 are 244 μW, 1.5 mW, and 2.5 mW @ 120 mW pump. Here, in order to avoid a CW peak on the optical spectrum, the highest pump power that can obtain a single-pulse operation is not chosen, because in some cases, the CW component in the spectrum may cause a slight increase in the intensity noise [42].

**Figure 2:**
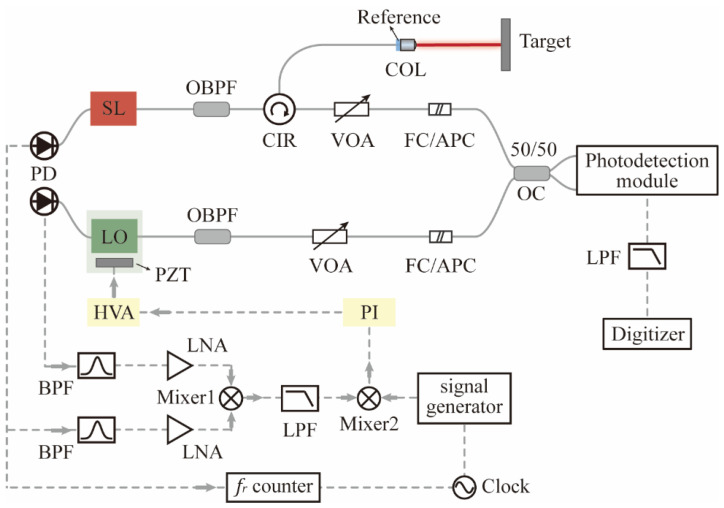
Experimental setup of absolute ranging using linear optical sampling. BPF, radio frequency bandpass filter; FC/APC, ferrule contactor/angled physical contact; HVA, high voltage amplifier; LNA, radio frequency low noise amplifier; LPF, radio frequency low-pass filter; OBPF, optical fiber bandpass filter; PI, proportional-integral controller; VOA, variable optical attenuator.

A high-reflective mirror mounted on a piezoelectric transducer (PZT) is used instead of the optical fiber mirror as the reflection arm of the LO laser to achieve phase locking. To obtain environmentally stable output characteristics, the SL adopts an all-PM fiber configuration, and no active control of the cavity length is applied. The differential repetition rate between the two lasers is stabilized by an external radio frequency (RF) signal source and the phase-locking scheme is shown in Figure 2. The repetition rates of the two lasers are photo-detected and the 9th harmonics are filtered out by bandpass filters [43]. Then, the two repetition rate harmonics are amplified and the differential frequency multiplied by 9 times is obtained at the output of an RF mixer. This signal is further mixed with a 22.20 kHz signal produced by an RF signal generator referenced to an Rb clock. The error signal is regulated by a loop filter, amplified, and applied to the PZT, thus closing the loop.

### 2.2. Design of Distance Measurement Setup

**Figure 3:**
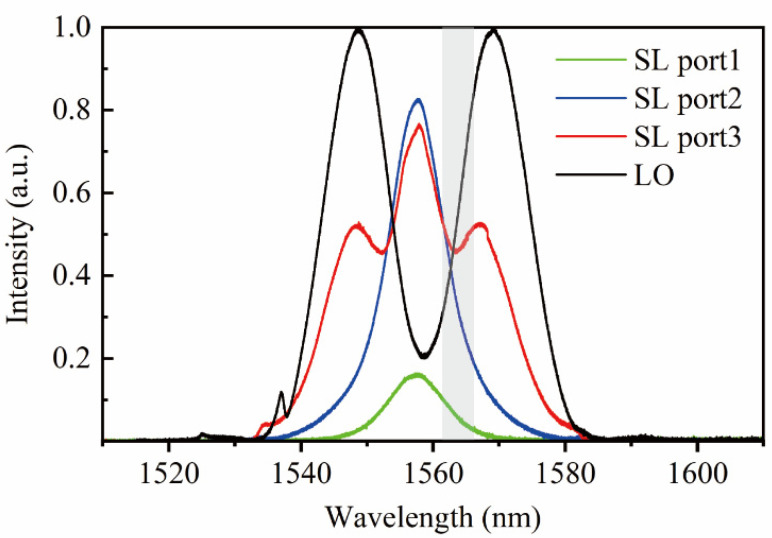
Optical spectrum of lasers (the passband of the filter is represented by the gray area).

Figure 2 illustrates the experimental setup of dual-comb absolute measurement using a triple-output all-PM signal laser. The ranging setup is based on a standard Michelson interferometer structure with a reference arm and a target arm. The cooperative target to be tested is fixed at a distance of ~100 mm, and the end face of the optical fiber jumper with a coating of 90% transmittance acts as the reference. The transmitted pulse train is collimated by a fiber collimator (Thorlabs, F220FC-1550, Newton, NJ, USA), and retroreflected laser pulses are received by the same collimator. A variable optical fiber attenuator (VOA) is used to control the output power to avoid photodetector saturation. The input power into the three ports is adjusted to a similar level to ensure a comparable RIN measurement. Figure 3 is the normalized optical spectrum of each output of the signal laser with 0.02 nm resolution. A pair of ~4 nm optical fiber bandpass filters are added to avoid aliasing the signal, and the passband is marked by a grey block. The linear optical sampling process occurred in a 2 × 2 optical coupler with a splitting ratio of 50/50. The photodetection module either includes a single avalanche photodetector (Thorlabs, APD430C/M) or a pair of balanced photodetectors (Thorlabs, PDB425C). During the entire ranging process, the mode-locked lasers are placed inside a vibration isolation box. The ranging signals are collected and processed by a commercial digitizer (National instrument, PXIe-5122, Austin, TX, USA) after low-pass filtering.

### 2.3. Intensity Noise of Signal Laser Output

**Figure 4:**
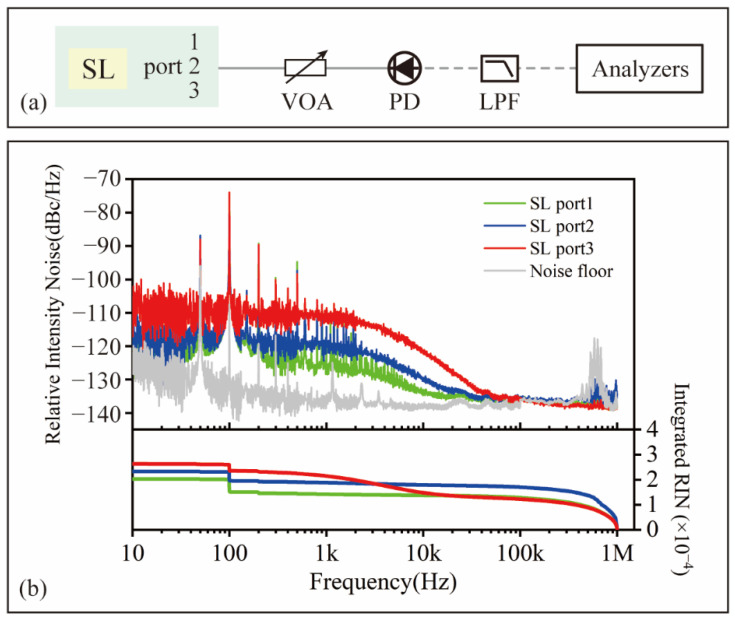
(**a**) Relative intensity noise measurement setup; (**b**) relative intensity noise power spectral density of the optical pulses from signal laser outputs.

The relative intensity noise of each port of the triple-output signal laser is characterized using the measurement setup shown in Figure 4a. The power-attenuated pulse train from different ports is photo-detected in an amplified detector (Thorlabs, PDA05CF2) and the power spectra are characterized by an FFT analyzer (SRS, SR770) at an acoustic frequency (10 Hz, 100 kHz) and an RF spectrum analyzer (RIGOL, DSA815) above 100 kHz. Figure 4b represented the measured RIN power spectral density (PSD). The corresponding relationship between the port number and the colorized curve is shown in the legend. Compared with ports 1 and 2, port 3 shows a significantly higher noise level (10 dB higher than the other ports from 100 Hz to 10 kHz), which contains amplified spontaneous emission (ASE) noise directly extracted from both directions in the nonlinearly amplified fiber loop. Port 2 (output of clockwise-running pulses) shows a higher RIN level than port 1 (output of counterclockwise-running pulses), while the difference is within 5 dB. The laser pulses from port 2 show a higher noise level because the clockwise-running pluses acquire more gain, thus higher ASE noise. The integrated value of the RIN of output ports 1, 2 and 3 str 0.020%, 0.023% and 0.026%. RIN measurement is limited by the noise floor when the frequency band is above 100 kHz. The RIN level of the laser is dominated by the pump diode technical noise and ASE noise [44,45].

### 2.4. Comparison of Distance Measurement Results

**Figure 5:**
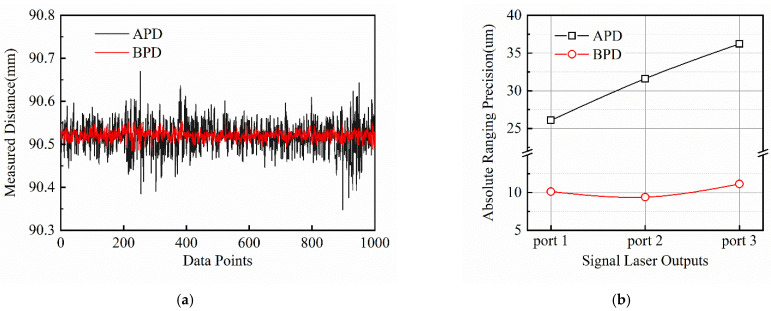
(**a**) Measured distance results in ~0.5 s; (**b**) Absolute ranging precision of the measured distance at a close target-reference separation.

A set of distance values measured within ~0.5 s from port 3 is shown in Figure 5a, and the Allan deviation without time averaging is calculated to characterize the absolute ranging precision shown in Figure 5b. The black and red traces represent the results using the avalanche photodetector and the balanced photodetector, respectively.

When the avalanche photodetector is used for detection, with the increase in laser output intensity noise, the ranging standard deviation of measurement using different outputs increases with the RIN of the output. Using ports 1, 2 and 3 for absolute ranging, the precision is 26.08393 μm, 31.60821 μm and 36.21884 μm, respectively, within the acquisition time of ~0.5 s. The balanced photodetector can be used to eliminate the impact of RIN on ranging precision. In the same acquisition time, the ranging precision using each port is 10.13781 μm, 9.10924 μm and 11.14095 μm, much smaller compared with a single photodetector. Note that, in our past work [46], we demonstrated that the three ports of this NALM mode-locked signal fiber laser have substantially the same timing jitter level. Therefore, the difference in the ranging results is independent of the quantum-limited timing jitter of the signal laser.

## 3. Dual-Comb Ranging Using Nonlinear Asynchronous Optical Sampling

The optical power level of the triple-output SL laser used in the above experiments cannot directly meet the requirements of the ASOPS principle. It is also better not to add an external EDFA, which may introduce more intensity noise. Therefore, we studied the impact of laser RIN on the dual-comb ranging precision based on nonlinear ASOPS in an independent experiment.

### 3.1. Design of Dual-Comb Ranging Setup

**Figure 6:**
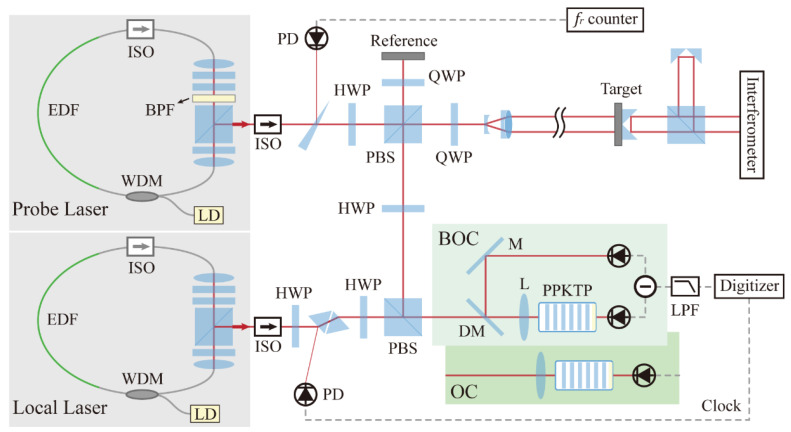
Experimental setup of absolute distance measurement using nonlinear ASOPS. BPF, optical bandpass filter; DM, dichroic mirror; HWP, half-wave plate; ISO, isolator; LPF, low pass filter; M, mirror; PBS, polarization beam splitter; PD, photodetector; QWP, quarter-wave plate; WDM, wavelength division multiplexer.

**Figure 7:**
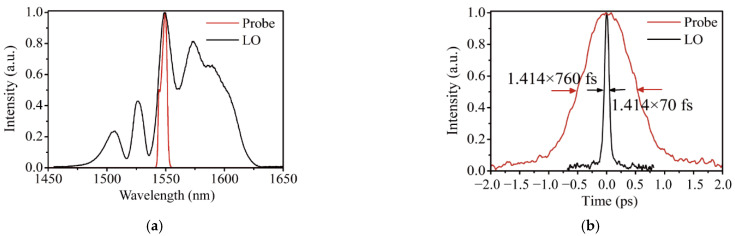
(**a**) Optical spectrum of the two nonlinear polarization evolution (NPE) lasers; (**b**) autocorrelation traces of the two NPE lasers.

The experimental setup is illustrated in Figure 6. A pair of homemade, almost identical nonlinear polarization evolution (NPE) mode-locked fiber lasers are used. The lasers operate in a stretched pulse regime [47] with close to zero cavity dispersion. The repetition rates of the probe laser and local laser are 99.991 MHz and 99.993 MHz, with a difference of roughly 2 kHz. The output optical spectra and autocorrelation traces from the two lasers are summarized in Figure 7. The LO is designed to direct output pulses close to the pulse duration transition limit [48,49]. The residual chirp is compensated by an added SF14 prism pair out of the cavity. The de-chirped pulse duration is 70 fs. The LO laser output average power is 45 mW @ 480 mW pump. To meet the Nyquist condition for sampling, we inserted an 8 nm bandpass filter centered at 1550 nm into the probe laser. The pulse dynamics of the probe laser are correspondingly switched from stretched-pulse to self-similar formation [50]. The probe laser output pulse duration is 760 fs and the output average power is 40 mW @ 580 mW pump. Note that inserting a bandpass filter outside the laser cavity is intentionally avoided here, to avoid the introduction of significant laser power attenuation, thereby limiting the signal-to-noise ratio (SNR) in ranging detection.

### 3.2. Measurement Result Based on OC or BOC

**Figure 8:**
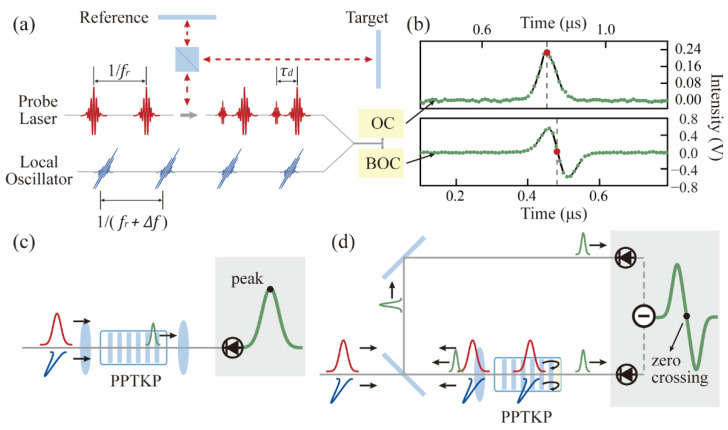
(**a**) Schematic setup of dual-comb absolute distance measurement using nonlinear ASOPS. (**b**) Ranging signal obtained in real-time. (**c**) Optical cross-correlator-based nonlinear ASOPS. (**d**) Balanced cross-correlator-based nonlinear ASOPS.

The measurement principle based on nonlinear ASOPS is shown in Figure 8a. A probe laser emits a femtosecond pulse train with a repetition rate of $f_{r}$. The retro-reflected laser pulses are combined and subsequently sampled by a local pulse train with a slightly different repetition rate of $f_{r}+\Delta f_{r}$ and orthogonal polarization. The principle of obtaining the ranging signal using an optical cross-correlator with a single photodetector is shown in Figure 8c. Intensity cross-correlation is also functioned by sum frequency generation (SFG) in a piece of type 2 phase-matched periodically poled KTiOPO4 (PPKTP). Note that there is no fringe structure in the obtained cross-correlation signal, therefore, we do not need a phase-locked repetition rate difference, which is necessary to stabilize the optical fringe pattern. In fact, free-running mode-locked lasers are routinely used in a nonlinear ASOPS scheme [28]. Therefore, the two NPE mode-locked fiber lasers used in this experiment are also free running. The two lasers share the same breadboard, which helps to counteract relative repetition rate drift caused by mechanical vibration.

The reference mirror and the objective mirror are also fixed at a short distance. The combined beam is directed into the OC, where the reference and target pulses are all optically sampled by the much shorter LO pulses. The OC signal is received by an avalanche photodetector. The output voltage signal from the photodetector is digitized and stored by a digitizer (National Instrument, Austin, TX, USA, PXIe-5122). The timing of the reference and target retro-reflected pulses is determined by the peak of the OC signal.

**Figure 9:**
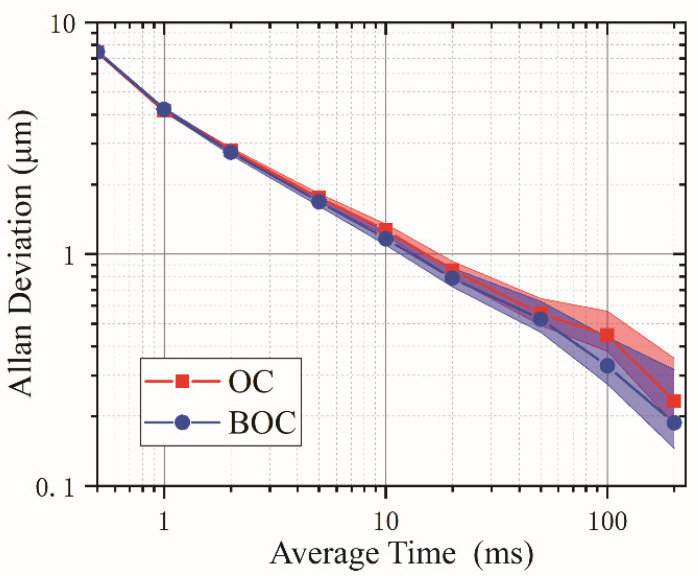
Allan deviation of the measured distance at 0.7-m target-reference separation.

The Allan deviation of the distance measurement with different averaging times at a fixed target mirror position ~0.7 m away is shown by the red trace in Figure 9. The target distance is obtained in real-time. The measurement precision is 7.436 μm at 0.5 ms averaging time, corresponding to the 2 kHz update rate. The precision reaches 554 nm at 50 ms averaging time and 232 nm at 0.2 s averaging time.

The BOC configuration is shown in Figure 8d. Essentially, it introduces two identical intensity cross-correlation paths with a stable time delay. The nonlinear crystal has a dichroic coating on the remote side, which transmits the sum frequency while reflecting the fundamental frequency. As a result, two identical SFG processes in a single crystal are realized. The measured BOC signal is also shown in Figure 8b. Instead of acquiring the signal peak, the zero-crossing of the BOC signal is used to determine the pulse TOF. The approach is similar to that of the OC scheme. Except for the replacement of the optical cross-correlator, other elements, such as the laser source and the fixed target to be measured, are the same.

The measurement result is shown by the blue trace in Figure 9. Comparably, the measurement precision is 7.447 μm at 0.5 ms averaging time, only ~11 nm difference from the result of OC. Note that this precision is at a similar level to the LOS-based ranging performance using balanced photodetection, as shown in Figure 5b. This means that both the standard ASOPS and LOS approach can achieve comparable ranging precision in dual-comb ranging applications. The precision in Figure 9 reaches 525 nm at 50 ms averaging time, ~29 nm lower than the OC result, and 187 nm at 0.2 s averaging time. The Allan deviation satisfies the square root of the mean time, which implies that white noise is the dominating noise source. This scatter is mainly dominated by the limited signal-to-noise ratio in the detection of the BOC signal and the error of zero-crossing extraction. When the average time is within 2 ms, the traces almost overlap, which indicates that the BOC does not outperform the OC in terms of measurement precision. Only above 50 ms averaging time, the BOC method shows better precision, while the improvement hardly exceeds 100 nm.

## 4. Discussion and Conclusions

In conclusion, this paper studies the impact of laser intensity noise on the dual-comb absolute ranging precision. For LOS, a triple-output mode-locked laser with different RIN levels acts as the signal laser. It is phase-locked with an LO such that the differential repetition rate is fixed. In a dual-comb ranging experiment, ranging precision deteriorates with increasing output laser RIN level, and the obtained ranging precision is 26.1 μm, 31.6 μm and 36.2 μm in the presence of RIN levels of 0.020%, 0.023%, 0.026%, respectively. By using balanced detection, the ranging precision in each port reaches 10.1 μm, 9.1 μm and 11.1 μm, respectively, improved significantly compared with a single photodetector. While, for ASOPS, there is no significant difference in measurement precision based on OC and BOC (7 μm Allan deviation without time averaging) even when free-running dual-comb sources are used. The difference between LOS and ASOPS lies in the TOF extraction algorithm. For LOS, laser intensity noise may distort interferometric beat notes and even fail the Hilbert transform algorithm, which is necessary to separate the envelope and the interferometric fringes. Therefore, balanced detection is beneficial to measurement precision improvement. While, for ASOPS, the nonlinear optical cross-correlation process technically avoids the optical fringes and makes the following TOF acquisition (either by center-of-gravity or zero-crossing determination) straightforward. The slight distortion of the signal caused by laser RIN hardly affects the single measurement precision and the BOC method is not necessary. This study provides a new avenue towards precision optimization in various dual-comb absolute ranging systems.

## Data Availability

Not applicable.
